# Antifungal activity of dual combination of hydroxychavicol with commercialized agents against oral Candida species

**DOI:** 10.1186/s40064-016-3396-6

**Published:** 2016-10-03

**Authors:** Wan Harun Himratul-Aznita, Che Omran Nor-Zulaila, Khairuddin Nurul-Fatihah

**Affiliations:** 1 Department of Oral Biology and Craniofacial Sciences, Faculty of Dentistry, University of Malaya, 50603 Kuala Lumpur, Malaysia; 2 Department of Oral Biology and Biomedical Sciences, Faculty of Dentistry, University of Malaya, 50603 Kuala Lumpur, Malaysia

**Keywords:** *Candida*, Hydroxychavicol, Amphotericin B, Synergistic

## Abstract

*Candida* spp. is the most prevalent species causing systemic fungal infections. The effect of antifungal agents were screened in vitro and their synergism effect were determined between hydroxychavicol (HC) in association with commercialized antifungal drugs—amphotericin B (AMB), and 5-fluorocytosine (5-FC) alone and in combination against five different oral *Candida spp.* in their planktonic states at different ratio (1:1 v/v; 1:2 v/v and 2:1 v/v). In vitro susceptibilities of *Candida* spp. to HC and commercialized antifungal agents were investigated by broth microdilution method as described by Clinical and Laboratory Standards Institute M38-A2. The intensity of the interactions was evaluated by visual reading and spectrophotometric method in checkerboard microdiluton assay, and the nature of the interactions was assessed by fractional inhibitory concentration index. The minimum inhibitory concentration (MIC_50_) of HC, AMB and 5-FC alone against five different planktonic oral *Candida* spp. ranged from 240 to 120, 8 to 15, and 2 to 8 µg/mL respectively. Positive synergistic effect existed between HC and AMB at 1:1 ratio in all *Candida* spp. However, there was no synergy effect observed in the majority of *Candida* spp. for the combination of HC with 5-FC. The data of combination between HC with AMB may be useful in the treatment of systemic infections caused by oral *Candida* spp. instead of the combination of HC with 5-FC.

## Background

Invasive fungal infections, such as candidiasis, represent a public health problem of major importance. Since its discovery in 1839 by Langenbeck, the genus *Candida* has been shown to be the causative agent of many infections and represent a component of the normal flora in the oral cavity (Fridkin and Jarvis 1996). According to previous study, candidal adherence to mucosal surfaces is considered as a critical initial step in the pathogenesis of oral candidiasis (Bokor-Bratic 2008).

The high global incidence and prevalence of oral candidiasis may be attributed to an increasing usage of broad-spectrum antibiotics, cytotoxic, corticosteroids, and to a growing number of immuno-suppressed individuals as well as those with common endocrine disorders (such as diabetes mellitus) or severe nutritional deficiencies (Johnson et al. 2004). The increasing incidence of fungal infections without a satisfactory response to the current antifungal therapy and the slow development of new agents with novel mechanisms of action have produced significant interest on associations between antifungal agents (Hemaiswaryaa et al. 2008).

HC is the major phenolic component, isolated from the aqueous extract of *P. betle* L., leafand has been reported to exhibit antibacterial activities against oral cavity pathogens (Sharma et al. 2009) by inhibit the growth and disrupt the permeability barrier of microbial membrane structures. However, the report on its antifungal activity is lacking.

Polyenes such as AMB (isolated from *Streptomycin* spp.) bind to ergosterol and disrupt the major lipid component of the fungal cell membrane. From 1950s until the discovery of the azoles, polyenes antifungal agents represented the standard of therapy for systemic fungal infections (Sugar 1986). While, flucytosine (5-FC) is a synthetic antimycotic compound and possess no intrinsic antifungal capacity. It will be converted into 5-fluorouracil (5-FU) once been taken up by susceptible fungal cells, and is further converted to metabolites that inhibit fungal RNA and DNA synthesis (Vermes et al. 2000).

Some of the most effective antifungal drugs are too toxic for continuous use or can only be administered intravenously (Ghannoum and Rice 1999). The ideal antifungal drug would be non-toxic, fungicidal, and amenable to self-administration. Combination therapy is one approach that can be used to improve the efficacy of antimicrobial therapy. Thus, the present study was carried out with the aim of investigating the combination of HC with AMB and 5-FC against planktonic of oral associated *Candida* spp.

## Methods

### Candida strains and growth condition

Five strains of *Candida* spp. used in this study were purchased from The American Type Culture Collection (ATCC), USA. The species were *C. albicans* ATCC 14053, *C. tropicalis* ATCC 13803, *C. parapsilosis* ATCC 22019, *C. lusitaniae* ATCC 64125 and *C. dubliniensis* ATCC MYA-2975. Upon revival, each respective *Candida* strain was stored in 20 % v/v glycerol and kept in −70 °C. All strains were also subcultured monthly on Yeast Peptone Dextrose (YPD) agar media (BD Difco, USA) and maintained weekly at 4 °C throughout the experimental period. Purity of the phenotypes was further confirmed by API Yeast Identification System (API 20C Aux, BioMerieux, France). Throughout the experiment period, the cultures were maintained on YPD agar up to a maximum of 2 weeks at 4 °C. Regular sub-culturing was carried out every 2 weeks to maintain viability of the cells.

### Preparation of the standard candida cell suspension, bioactive compound and antifungal agents

Each *Candida* strain was respectively cultured on Yeast Peptone Dextrose (YPD) agar media (BD Difco, USA) at 37 °C for 24 h according to Harun et al. (2014). The turbidity of the suspension was adjusted to an optical density (OD_550nm_) of 0.144 which is equivalent to 1 × 10^6^ cells/mL. HC was isolated in the pure form from the chloroform extraction of the aqueous leaf extract of *P. betle* L., (Piperaceae) as described previously (Sharma et al. 2009). AMB and 5-FC were obtained as standard liquid and powders purchased from Sigma Chemical Co. (St. Louis, MO).

A stock solution of 1000 µg/mL HC and 250 µg/mL AMB was prepared in 5 % DMSO and stored at 4 °C until used. A 5 % DMSO did not influence the growth and viability of fungi in a pilot study carried out prior to the experiment in our laboratory. A stock solution of 250 µg/mL of 5-FC was prepared in sterile distilled water. AMB was also used in the study as the positive control.

### Determination of MIC

The minimum inhibitory concentrations (MIC) of active compound and antifungal agentsalone against *Candida* spp. was determined according to the microdilution method of the CLSI M38-A2using 96-well microtiter plates with some modifications (Clinical and Laboratory Standards Institute 2008). This modification involved the usage of YPD instead of Sabaroud Dextrose Agar (SDA) or Potato Dextrose Agar (PDA) as describe in Harun et al. (2014). Concentration of ½ MIC was used throughout the study as the research was focused in controlling the population of *Candida* spp. in the oral cavity rather than giving a complete killing effect, considering *Candida* spp. as oral commensals. A broth microdilution method was recommended by CLSI as a general standard methodology for testing active compound or commercialized antifungal agent. Thus, this method was employed to analysed the MIC of HC, AMB and 5-FC.

Each well contained the 10 µL of *Candida* spp. at a final concentration of 1.0 × 10^6^ cells/mL, 100 µL of YPD broth, and 100 µL of HC, AMB and 5-FC. YPD broth without test agents was included as an agent-free control, and YPD broth was used as medium blank. All plates were incubated in an aerobic incubator at 37 °C for 24 h, after which the growth was determined spectrophotometrically at 550 nm by means of a microplate reader (PowerWave 200, Bio-Tek Instruments, and Winooski, VT, USA). The data were reported as the median of at least 3 independent tests.

### Assessment of HC and commercialized antifungal agents against Candida spp

Synergistic effects of HC to AMB and HC to 5-FC at a ratio of 1:1 v/v, 1:2 v/v and 2:1 v/v against five planktonic of oral *Candida* spp. were quantitatively determined using the checkerboard microdilution method as described in previous study (Cuenca-Estrella 2004).

Briefly, 1000 µL HC, 250 µL AMB and 250 µL5-FC were used as initial concentration in this study. A 1:1 ratio of either HC and AMB or HC and 5-FC was prepared. Firstly, 50 µL of HC was added into well 1 of 96 microtitre plate. Followed by an addition of 50 µL of either AMB. It was then serially diluted up to well 12. Inoculum of 1 × 10^6^ cells/mL was added to each well and the plates were incubated at 37 °C for 24 h and read spectrophotometrically at 550 nm using a microplate reader (PowerWave 200, Bio-Tek Instruments, and Winooski, VT, USA). Negative control was prepared without the addition of HC and AMB. Similar procedure was carried out for 1:1 ratio of HC and 5-FC. The analysis was also performed with 1:2 and 2:1 ratio of HC:AMB and HC:5-FC.

The MIC combination was determined as the concentrations of antifungal drugs that resulted a 50 % reduction in absorbance compared to that of control. The MIC values of combined HC with AMB and 5-FC used to determine the fractional inhibitory concentration (FIC). The FIC index (∑FIC, the sum of individual FICs) was calculated using the formula: ∑FIC = MIC (A_comb_)/MIC (A_alone_) + MIC (B_comb_)/MI7C(B_alone_).

Two drugs or bioactive compounds are defined as having synergistic effect, if the FIC indexes are ≤0.5, whereas they are said to have indifference when the FIC 0.5 but ≤4, and antagonistic when FIC index was >4 (Cuenca-Estrella 2004).

## Results

### MIC_50_ of HC, AMB and 5-FC against oral Candida spp

The inhibitory activities of HC, AMB and 5-FC against a series of oral-associated *Candida* spp. were investigated (Table 1). Planktonic growth of *Candida* spp. was susceptible to different antifungal drugs at varying concentrations. The MIC_50_ range of HC against *Candida* spp. after 24-h incubation was subsequently 240–120 µg/mL. It has been found that, *C. parapsilosis* and *C. dubliniensis* were the most susceptible to HC.

**Table 1:** Susceptibilities of standard *Candida* spp. to HC alone and in combination with AMB and 5-FC (1:1) ratio

Species	MIC (µg/mL) alone^a^			MIC (µg/mL) combination (1:1) ratio^a^							
	HC	Amp B	5-FC	HC^b^	Amp B	HC + Amp B FICI	INT	HC^c^	5-FC	HC + 5-FC FICI	INT
*C. albicans*	240	8	2	2	0.5	0.07	SYN	8	2	1.03	IND
*C. parapsilosis*	120	8	2	4	1	0.16	SYN	15	4	2.04	IND
*C. tropicalis*	240	4	8	4	1	0.27	SYN	30	8	1.13	IND
*C. dubliniensis*	120	4	2	2	0.5	0.26	SYN	15	4	2.13	IND
*C. lusitaniae*	240	15	4	8	2	0.17	SYN	8	2	0.53	IND

MIC and FICI values are shown as a mean of three independent experiments

*ANT* antagonism, *IND* indifference, *SYN* synergy, *INT* interpretation

^a^The MIC end point is based on the lowest drug concentration producing a decrease of 50 % inhibition of fungal growth compared to untreated

^b^MIC of HC when in combination with AMB

^c^MIC of HC when in combination with 5-FC

The MICs of individual AMB and 5-FC against the test strains of *Candida* species were lower than those of HC, ranging from 8 to 15 and 2 to 8 µg/mL, respectively.

AMB alone was active against *C. tropicalis* and *C. dubliniensis* at MIC of 4 µg/mL, *C. albicans* and *C. parapsilosis* at MIC, of 8 µg/mL and *C. lusitaniae* at MIC of 15 µg/mL.

5-FC alone was active against *C. albicans, C. parapsilosis* and *C. dubliniensis* at 2 µg/mL of MIC concentration, while *C. lusitaniae* and *C. tropicalis* was slightly less susceptible to 5-FC at 4 and 8 µg/mL of MIC.

### The effect of HC/AMB on the planktonic growth of Candida spp

Checkerboard analysis, has determined that the combination of HC and AMB having MIC of synergistic effect against five planktonic *Candida* spp. with ∑FIC index ≥0.5. The FIC index for HC in combination with AMB was calculated as shown in Tables 1, 2 and 3. The combination HC/AMB at 1:1 ratio yielded synergism interaction against all five *Candida* species, while at 1:2 and 2:1 ratio of combination, *C. tropicalis* and *C. lusitaniae* showed indifferent effect.

**Table 2:** Susceptibilities of standard *Candida* spp. to HC alone and in combination with AMB and 5-FC (1:2) ratio

Species	MIC (µg/mL) alone^a^			MIC (µg/mL) combination (1:2) ratio^a^							
	HC	Amp B	5-FC	HC^b^	Amp B	HC + Amp B FICI	INT	HC^c^	5-FC	HC + 5-FC FICI	INT
*C. albicans*	240	8	2	4	1	0.14	SYN	15	4	2.06	IND
*C. parapsilosis*	120	8	2	4	1	0.16	SYN	8	2	1.06	IND
*C. tropicalis*	240	4	8	15	4	1.06	IND	30	8	1.13	IND
*C. dubliniensis*	120	4	2	4	1	0.29	SYN	8	2	1.06	IND
*C. lusitaniae*	240	15	4	30	8	0.67	IND	15	4	1.06	IND

MIC and FICI values are shown as a mean of three independent experiments

*ANT* antagonism, *IND* indifference, *SYN* synergy, *INT* interpretation

^a^The MIC end point is based on the lowest drug concentration producing a decrease of 50 % inhibition of fungal growth compared to untreated

^b^MIC of HC when in combination with AMB

^c^MIC of HC when in combination with 5-FC

**Table 3:** Susceptibilities of standard *Candida* spp. to HC alone and in combination with AMB and 5-FC (2:1) ratio

Species	MIC (µg/mL) alone^a^			MIC(µg/mL) combination (2:1) ratio^a^							
	HC	Amp B	5-FC	HC^b^	Amp B	HC + Amp B FICI	INT	HC^c^	5-FC	HC + 5-FC FICI	INT
*C. albicans*	240	8	2	2	0.5	0.07	SYN	30	8	>4	ANT
*C. parapsilosis*	120	8	2	2	0.5	0.08	SYN	15	4	2.25	IND
*C. tropicalis*	240	4	8	8	2	0.53	IND	30	8	1.12	IND
*C. dubliniensis*	120	4	2	4	1	0.28	SYN	30	8	>4	ANT
*C. lusitaniae*	240	15	4	8	2	0.17	SYN	15	4	1.06	IND

MIC and FICI values are shown as a mean of three independent experiments

*ANT* antagonism, *IND* indifference, *SYN* synergy, *INT* interpretation

^a^The MIC end point is based on the lowest drug concentration producing a decrease of 50 % inhibition of fungal growth compared to untreated

^b^MIC of HC when in combination with AMB

^c^MIC of HC when in combination with 5-FC

At 1:1, 1:2 and 2:1 ratio of HC/AMB against *C. albicans*, the MIC of HC were decreased up to 120- to 60-fold, while MIC of AMB were decreased about 8- to 16-fold. As shown in Table 1, strong synergistic effect has been demonstrated at 1:1 and 2:1 ratio with the FIC index of 0.07.

Against *C. parapsilosis*, strong synergistic effect has been observed at 2:1 ratio of HC/AMB with the FIC index of 0.08, where the MIC combination was markedly decreased (60- to 4-fold respectively). However, at 1:1 and 1:2 ratios, the synergism effect was also observed with the FIC index of 0.16, which is slightly higher than those observed in 2:1 ratio.

As illustrated in Table 1, a synergistic effect was observed against *C. tropicalis* (FICI, 0.27) at 1:1 ratio, however showed indifferent effect in 2:1 (FICI, 0.53) and 1:2(FICI, 1.06) ratios. MIC of HC/AMB in 1:1 ratio has shown decreased of about 60-fold and 8-fold respectively (Table 1).

Against *C. dubliniensis*, the MIC of HC in association with AMB at 1:1 ratio decreased about 60:8-fold (FICI, 0.26) where synergism was observed. Differently, a synergism was also found at 1:2 and 2:1 ratio with FIC index of 0.29 and 0.28 where the MIC were decreased about 30:4-fold.

Against *C. lusitaniae,* it was found that a combination of HC and AMB exhibited strong synergism in 1:1 and 2:1 ratios with the FICI index of 0.17. The MIC of HC and AMB giving a synergistic effect was found to be 30 and 8- fold lower than the MIC alone respectively. In comparison, the lowest activity was displayed at 1:2 with the FIC index of 0.67.

### The effect of HC/5-FC on the planktonic growth of Candida spp

The MIC of the combination of HC/5-FC as well as the MICs of the single drug tested at the ratio of 1:1, 1:2 and 2:1, are given in Tables 1, 2 and 3. The FICI values ranged from 0.53 to 2.13 for the combination at 1:1 ratio, from 1.06 to 2.06 for 1:2 ratio and from 1.06 to >4 for 2:1 ratio.

As shown in Table 1, only *C. lusitaniae* of the combination of HC/5-FC in 1:1 ratio after 24-h incubation had partial synergistic properties with FIC index of 0.53. Indifferent interactions were observed in *C. albicans, C. parapsilosis*, *C. tropicalis* and *C. dubliniensis* with FIC index of 2.25 and 1.06 respectively.

All of *Candida* spp. showed indifferent reaction at 1:2. However, antagonism has been observed for *C. albicans* and *C. dubliniensis* with FIC index >4 at 2:1 ratio. The MIC of 5-FC against *C. albicans* and *C. dubliniensis* increased up to fourfold. Our data showed 5-FC alone demonstrated greater effectiveness than in combination with HC against all *Candida* spp.

## Discussion

Treating *Candida* infections with monotherapy is becoming more difficult, a major problem being the emerging drug resistance during treatment with various conventional antifungal agents. The mechanisms of antifungal resistance are categorized as primary or secondary and are related to intrinsic or acquired characteristics of the fungal pathogen, including interference with the antifungal mechanism of the respective drug or the decrease in target drug levels (Akins and Sobel 2009). Hence, there is a need to search or develop new formulation by using combinations as alternate and effective antifungal agents that have few or no side effects. The selection of the ATCC reference strains *C. albicans*, *C. parapsilosis*, *C. tropicalis*, *C. dubliniensis,* and *C. lusitaniae* was based on various reports of the prevalence of *Candida* species in the oral cavity (Harun et al. 2014). Although the reference strains were isolated originally from blood, similar strains have also been reported to be present in the oral cavity (Harun et al. 2014).

In recent years, the use of natural compound in combination with conventional antifungal agents to achieve drug synergy has attracted much attention. Natural compound with high effectiveness and fewer side effects are desirable as substitutes for chemical treatments which have various adverse effects. *Piper betle* L., (Piperaceae) has been extensively used in traditional herbal remedies in India, China, Taiwan, Thailand and many other countries. It is reported to possess various pharmacological activities such as antimicrobial, antioxidant; antimutagenic; anticarcinogenic; anti-inflammatory and etc. (Hemaiswaryaa et al. 2008). *Piper betle* was reported as good anticandidal agent since it contains bioactive components such as 4-chromanol (Kawsud et al. 2014), allylpyrocatechol (Dwivedi and Tripathi 2014), hydroxychavicol (Dwivedi and Tripathi 2014) and others.

HC has been reported to have antifungal activity. Although it is a useful natural compound for treating fungal infections, its high MIC prevents its effective use in clinical study. Therefore, it will be more effective to use HC in combination with conventional antifungal agents rather than used alone. To our knowledge, the in vitro phenomenon of synergism of HC with AMB and 5-FC against *Candida* spp. is reported here for the first time.

There are many models for experimental designs to measure such combination effects. One of the best known and very simple forms of such tests is the ‘chequerboard’ experiment in which a two dimensional array of serial concentrations of test compounds is used as the basis for calculation of a fractional inhibitory concentration index (FICI) (William et al. 2003). Applying these ideas to in vivo and clinical investigations of combination antifungal therapy is difficult, and no standards for interpretation of these data have been recommended. Analysis and comparison of results across in vivo and clinical studies requires careful consideration of the nature of pathogen, host, host immune status, study design, and study endpoints (Melissa et al. 2004). The effects observed in these models will not precisely apply to all aspects of the clinical. However, in vitro testing might suggest an effective antifungal at which the dose needed to achieve the desired effect. They also represent the best-controlled data we have and, upon review, they help us gain a better understanding of how these drugs might behave when used together. They also represent the best-controlled data we have and, upon review, they help us gain a better understanding of how these drugs might behave when used together. Thus, the only way to resolve some of these issues is to use the available in vitro and in vivo data to drive the design of carefully selected clinical studies of combination therapy in patients.

Combination between HC and AMB at 1:1, 1:2 and 2:1 ratio has resulted synergistic against most of species tested. While combination of HC with 5-FC at 1:1, 1:2 and 2:1 ratio has resulted indifferent and antagonistic interaction. Combinationat 1:1 ratio between HC with AMB considered as a good antifungal combination since it showed synergistic effect at lowest ratio used in this study against *Candida* spp.

In the search for the mechanism of synergism between HC with AMB, their individual antifungal modes of action need to be considered. From previous study, they reported that, HC alters the cell membrane structure, resulting in the disruption of the permeability barrier of microbial membrane structure (Nalina and Rahim 2007). While, polyenes antifungal are able to bind to ergosterol which is the main sterol in the fungal cell membrane. Enzymes in the ergosterol pathways are the targets of many antifungal agents. AMB binds with ergosterol, a component of fungal cell membranes, forming pores that cause rapid leakage of monovalent ions and subsequent fungal cell death (Mesa-Arango et al. 2012).

More importantly, our results showed that addition of HC to AMB could possibly enable reduced dosages of both agents and thus potentially reduced drug-associated toxicities which are frequently observed at the high dosages of using AMB alone (Van’t Hof et al. 2000). Although the exact mechanism of interaction between HC and AMB is not known, it is possible that the simultaneous inhibition of different fungal cell targets occur. Combination of HC/AMB appears to involve the disruption of cell membrane structure that cause rapid leakage of monovalent ions allows the passage of one or both agents. Thus, the synergism effect was observed in this combination.

5-FC resistant strains of *Candida* being clinically significant, thus there is a need for the development of new therapeutic agents especially in combination studies (Bondaryk et al. 2013). The results of these study provide evidence of antagonism effect between the combination of HC with 5-FC, and suggested that these agents should not be co-administered. A detailed mechanism explanation why the combination of HC and 5-FC antagonistic is unknown. It also can be suggested that it might be related to the disruption and changes in fungal cell membrane function due to the effect of HC at first place. According to Patel (1998), 5-FC is used in combination with other antifungal, such as AMB rather than used as monotherapy due to the increasing of drug resistance in combating fungal pathogens. In vitro data regarding the combination of both drugs against *Candida* species are numerous and somewhat contradictory showing antagonistic (Vandeputte et al. 2012).

## Conclusion

In conclusion, HC has antifungal activity but a very high MIC value against oral *Candida* spp. However, the combination of HC with AMB exhibited a synergistic activity against all tested *Candida* species. Whereas, in combination with 5-FC exhibited indifference and antagonism activity. Therefore, the combination of HC/AMB effective at treating fungal infections and might be promising for future research into the pharmacological aspect. However, in vivo testing needs to be performed to support these findings.
